# The impact of a postoperative multimodal analgesia pathway on opioid use and outcomes after cardiothoracic surgery

**DOI:** 10.1186/s13019-022-02067-3

**Published:** 2022-12-30

**Authors:** Ceressa T. Ward, Vanessa Moll, David W. Boorman, Lijo Ooroth, Robert F. Groff, Trent D. Gillingham, Laura Pyronneau, Amit Prabhakar

**Affiliations:** 1 Convergent Genomics, 425 Eccles Avenue, South San Francisco, CA 94080 USA; 2 Department of Anesthesiology, Emory University School of Medicine, Atlanta, GA USA; 3 Mercer University College of Pharmacy, Atlanta, GA USA; 4 Office of Quality, Emory Healthcare, Atlanta, GA USA; 5 CVS Pharmacy, Douglasville, GA USA; 6 Potrero Medical, Hayward, CA USA

**Keywords:** Cardiac surgery, Enhanced recovery after cardiac surgery, Multimodal analgesia (MMA), Opioid, Postoperative analgesia

## Abstract

**Objective:**

The Enhanced Recovery after Surgery Cardiac Society recommends using multimodal analgesia (MMA) for postoperative pain however, evidence-based guidelines have yet to be established. This study examines the impact of a standardized postoperative MMA pathway in reducing opioid consumption and related complications after cardiothoracic surgery (CTS).

**Methods:**

Within a multicenter healthcare system, a postoperative MMA pathway was developed and implemented at two CTS intensive care units (ICU) while the other CTS ICU opted to maintain the existing opioid-based pathway. A retrospective chart review was conducted on patients admitted to a CTS ICU within this healthcare system after conventional coronary artery bypass grafting and/or valve surgery from September 1, 2018, to June 30, 2019. Comparative analysis was conducted on patients prescribed MMA versus those managed with an opioid-based pathway. The primary outcome was total opioid consumption, converted to morphine milligram equivalents, 72-h post-surgery. Secondary outcomes included mobility within one-day post-surgery, ICU length of stay (LOS), time to first bowel movement (BM), and time to first zero Richmond Agitation-Sedation Scale (RASS).

**Results:**

Seven hundred sixty-two adults were included for final analysis. The MMA group had a higher body mass index, higher percentage of females, were more likely classified as African American and had higher scores for risk-adjusted complications. General Linear Model analysis revealed higher opioid consumption in the MMA group (Est. 0.22, *p* < 0.0009); however, this was not statistically significant after adjusting for differences in fentanyl usage. The MMA group was more likely to have mobility within one-day post-surgery (OR 0.44, *p* < 0.0001), have longer time to first BM (OR 1.93, *p* = 0.0011), and longer time to first zero RASS (OR 1.62, *p* = 0.0071). The analgesia groups were not a predictor for ICU LOS.

**Conclusions:**

Opioid consumption was not reduced secondary to this postoperative MMA pathway. The MMA group was more likely to have mobility within one-day post-surgery. Patients in the MMA group were also more likely to have prolonged time to first BM and first zero RASS. Development and evaluation of a perioperative MMA pathway should be considered.

**Supplementary Information:**

The online version contains supplementary material available at 10.1186/s13019-022-02067-3.

## Introduction

Acute pain after surgery is typically of moderate to severe intensity, most notably during the first 48-h. After cardiac surgery, procedural trauma from a sternotomy, sternal retraction, radial artery/saphenous vein harvesting, and chest tube insertion necessitates sufficient analgesia to alleviate noxious stimuli without impeding recovery. Historically, postoperative pain management has been solely reliant on opioid administration [1, 2]. Although opioids have the advantages of being effective, relatively inexpensive, and available in various dosage forms, adverse effects commonly associated with opioids such as sedation, respiratory depression or ileus, can delay and confound postoperative recovery [1, 3].

Opioid abuse accounts for a staggering number of opioid-related morbidity and mortality in the United States. Among the theories regarding the origin of this epidemic, postoperative opioid use has been clearly identified as a contributory factor, and measures such as multimodal analgesia (MMA) have been implemented to reduce usage, especially in opioid-naïve patients [4, 5]. However, minimizing opioid utilization presents a conundrum for providers as inadequately controlled postoperative pain may lead to chronic pain syndrome, ongoing opioid use, increased morbidity, impaired functionality and quality of life, delayed recovery time, and higher healthcare costs [1, 6]. With > 80% of patients reporting inadequate pain relief, a need for optimization of postoperative pain management exists [6].

Multimodal analgesia employs a combination of analgesics (e.g., acetaminophen (APAP), gabapentinoids, ketamine, lidocaine, and non-steroidal anti-inflammatory drugs) and/or therapeutic techniques (e.g., regional anesthesia) to target different sensory pathways in the central and peripheral nervous system concomitantly, thereby minimizing postoperative opioid utilization and subsequent complications [5, 7–9]. Studies have also demonstrated that a reduction in opioid consumption correlates with an increase in mobility, a decrease in fatigue, and an overall decrease in symptom burden for patients in multiple surgical settings [1, 10]. Based on these documented benefits, the Enhanced Recovery After Surgery (ERAS) Cardiac Society strongly recommends the incorporation of a multimodal, opioid-sparing strategy; however, guidelines for MMA management utilizing non-opioid analgesics in the cardiac surgery population have yet to be defined [7, 8].

In accordance with ERAS recommendations, a postoperative MMA pathway was piloted at two of the three cardiothoracic surgery intensive care units (CTS ICU) within a multicenter healthcare system. We hypothesized that a MMA pathway would reduce opioid consumption and opioid-related postoperative complications compared to opioid-based pain management. The primary outcome was cumulative opioid consumption 72-h post-coronary artery bypass grafting (CABG) and/or valve surgery. Secondary outcomes for this study were the percentage patients with documented mobility within one-day post-surgery, ICU length of stay (LOS), time to first bowel movement (BM), and time to first zero Richmond Agitation-Sedation Scale (RASS).

## Methods

A postoperative MMA pathway was developed and implemented within a multicenter healthcare system at two CTS ICUs while the other CTS ICU opted to maintain an existing opioid-based pathway. A retrospective chart review was conducted to evaluate the effectiveness of this MMA pathway on reducing opioid consumption and subsequent complications. The Institutional Review Board approved this minimal risk study (IRB#00113517; approved July 31, 2019), including a waiver for written informed consent.

Utilizing the Society of Thoracic Surgeons (STS) Adult Cardiac Surgery Database (ASCD) version 2.9 [11], a report cross-referencing patients who underwent CABG and/or valve surgery during the specified timeframe was generated. A manual review of the electronic medical record was conducted on patients ≥ 18 years old who were admitted to a CTS ICU within this academic tertiary care health system between September 1, 2018 and June 30, 2019 for postoperative management after conventional CABG and/or valve surgery. Eligible patients were stratified to either the MMA or opioid-based group depending upon the analgesia pathway utilized at the admitting CTS ICU.

Patients were excluded if they were admitted to a CTS ICU after one of the following surgical procedures: transcatheter aortic valve replacement, minimally invasive valve repair or replacement, robotic-assisted valve repair or replacement, robotic-assisted CABG, or reoperation within 72-h of initial procedure. Patients were also excluded if they had a history of chronic liver disease (defined as elevated transaminases for at least six months, presence of cirrhosis, and elevated international normalized ratio), chronic kidney or end-stage renal disease, and/or opioid use for documented chronic pain.

### Postoperative analgesia pathways

Surgical patients arriving to the CTS ICU on an active infusion of analgosedation were transitioned to the respective analgesia pathway after extubation.

In the opioid-based pathway, scheduled oral APAP was standardized for all patients unless there was a contraindication to therapy. Other analgesics were prescribed at the discretion of the surgical and/or admitting critical care medicine team. For scheduled administration, prescribers had the option of intravenous (IV) ketorolac, lidocaine topical film, and/or pregabalin. Hydrocodone-APAP, oxycodone-APAP and/or IV morphine, at varying doses, were prescribed for as-needed administration.

Utilizing the MMA pathway, all patients were prescribed scheduled oral APAP, lidocaine topical film, and pregabalin in addition to IV hydromorphone and oxycodone that would be administered on an as-needed basis.

The two postoperative analgesia pathways are fully outlined in Table 1. None of the patients included in the study received regional anesthesia blocks.

**Table 1:** Postoperative analgesia pathways

*Conventional, opioid-based analgesia pathway*	
Standardized Analgesia Order	Acetaminophen (APAP) 650 mg PO every 6-h as needed for mild pain
Opioids Options (ordered at the discretion of the provider)	Hydrocodone-APAP 5 mg/325 mg PO every 4-h as needed for moderate pain^a^ Morphine IV every 1-h as needed for pain –1, 2 or 3 mg recommended for patients > 70 years old for specified pain severity –2, 4 or 6 mg recommended for patients < 70 years old for specified pain severity Oxycodone-APAP 5 mg/325 mg PO every 4-h as needed for specified pain severity
Non-opioid Options (ordered at 'the discretion of the provider)	Ketorolac 15 mg IV every 6-h for 3 doses Lidocaine topical film 1 or 2 patches every 24-h Pregabalin 75 mg capsule PO every 12-h for 4 days^b^
*Multimodal analgesia pathway*	
Standardized Analgesia Orders (unless contraindicated)	APAP 650 mg PO every 6-h for 3 days Lidocaine topical film 2 patches every 24-h Pregabalin 75 mg capsule PO every 12-h for 4 days^b^ Hydromorphone 0.5 or 1 mg IV every 6-h as needed for specified pain severity Oxycodone 5 mg or 10 mg PO every 4-h as needed for specified pain severity

Gabapentinoids were dose adjusted for renal dysfunction

*PO* Per Os (by mouth), *IV* intravenous

^a^For patients > 70 years old with moderate pain

^b^Patients were resumed on gabapentin if they were previously taking this therapy as an outpatient

### Data collection

The following demographics were retrieved from the STS ACSD version 2.9 [11]: age, sex, race/ethnicity, and body mass index (BMI). Adult cardiac surgery risk-adjusted outcomes for prolonged postoperative ventilation (PPV), operative mortality (OM), and operative major morbidity or mortality composite (OMM) were also recorded. These STS risk scores, calculated prior to surgery, classified the patient's probability of operative complications based on the patient’s preoperative medical history [12]. Calculated percentages for PPV and OM estimated the likelihood of mechanical ventilation needed for > 24 h and in-hospital death or any death within 30-days of the initial procedure, respectively. An OMM percentage predicted the likelihood of in-hospital death or death within 30-days of surgery or major morbidities such as prolonged ventilation, deep sternal wound infection, stroke, renal failure, or reoperations for bleeding or cardiac issues [13].

Charts were manually abstracted to obtain type of surgery, use of multimodal adjuncts, and total dosage of opioids administered within 72-h post-surgery. Cumulative opioid dosages were converted to oral morphine milligram equivalents (MME), using published opioid conversion factors, to allow for comparative analysis [14].

Patients with activated CTS order sets were imported into a dashboard, developed by the institution’s data analyst, and the following was collected: mobility within one-day post-surgery, time to first BM, time on ventilator, and time to first zero RASS. Mobility was defined as sitting at the edge of the bed with feet dangling, moving from bed to chair, and/or walking.

### Statistical methodology

All data were analyzed with SAS 9.4 (SAS Institute Inc. 2013, Cary, NC). Preliminary analyses were conducted using nonparametric tests to avoid assumptions of normality: Spearman Rank Correlation, Mann–Whitney U Test, Fisher Exact Test, Chi-Square Test, and Mantel–Haenszel Chi-Square Test. Full analyses used binary or multinomial logistic regression and the General Linear Model (GLM). Stepwise selection was used for multiple predictors in the same regression model.

### Adjusting for variations in fentanyl use

Patients that received ≥ 1000 mcg of IV fentanyl and had concomitant MME of ≥ 500 mg were identified. To neutralize the effect of any outlier patients who received fentanyl, the actual MME was replaced with the predicted MME had any of these patients been in the opioid group. This was deemed less biased than simply omitting any outlier patients from further analysis.

### Creating outcome sets

To mitigate the concern of false positives from analyzing too many outcome variables, closely correlated outcomes were omitted while loosely correlated outcomes were analyzed initially together, and if significant, were then analyzed separately. With these adjustments, there were still three separate outcome sets, so the alpha threshold to reject the null hypothesis was lowered to 1.7%, based on the Dunn–Šidák Correction (α_1_ = 1 − (0.95)^1/3^), restoring the effective rate of at least one false positive to 5% [15]. Predictors with *p* values between 0.017 and 0.05 were omitted for multiple regression, but the confidence intervals were kept at 95%.

Time on the ventilator was closely correlated to ICU LOS (Spearman Rank Correlation, *p* < 0.0001, r = 0.42); therefore, we omitted time on the ventilator (Additional file 21). Time to first zero RASS and first BM were significantly correlated with ICU LOS. However, they were not significantly correlated to each other. These three variables were dichotomized and then analyzed as a multinomial set with 8 groups: ICU LOS split at 60-h, time to first BM 0–3 days versus 4–9 days, and time to first zero RASS 0 days versus 1–2 days. ICU LOS ≤ 60-h, first BM at 0–3 days and first zero RASS at 0 days were used as the reference group. These variables were analyzed initially as a set and then separately if significant differences were noted. Neither MME nor documented mobility were significantly associated with other outcomes, so they were analyzed separately. See Additional file 21.

## Results

### Demographics

**Fig. 1:**
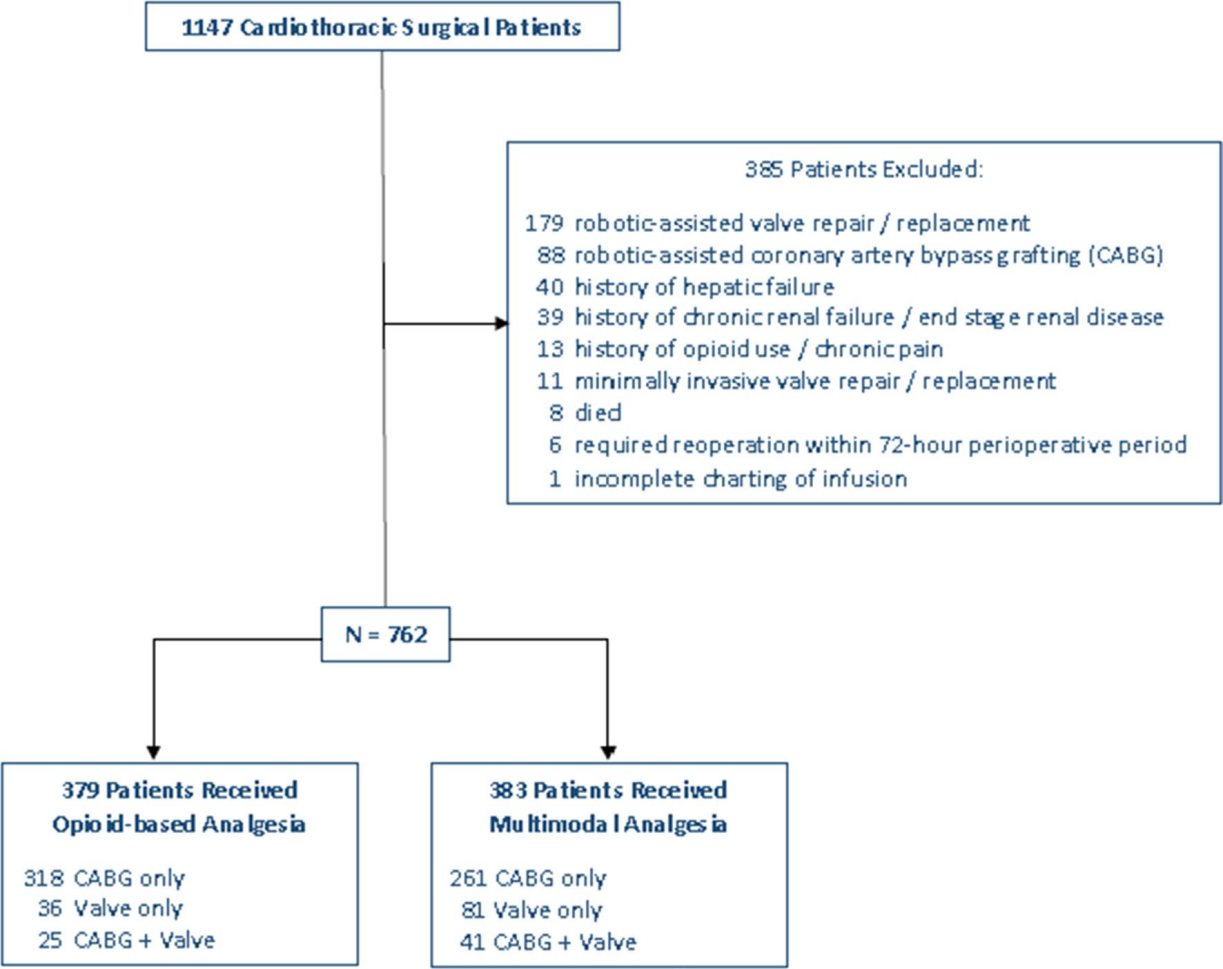
Inclusion flow chart

Between September 1, 2018, and June 30, 2019, 1147 cardiac surgeries were conducted at this academic tertiary care health system with 762 patients included for final analysis (Fig. 1).

Descriptive statistics for demographics and confounders are provided in Table 2. Patients in the two analgesic groups (MMA versus opioid-based) were similar in age (*p* = 0.095). The MMA group had a higher percentage of females (*p* = 0.0035), higher BMI (*p* = 0.0006), and were more likely classified as African American (*p* = 0.0017). As evidenced by the calculated preoperative adjusted-risk scores (PPV, OMM, and OM), patients in the MMA group were at significantly higher risks of postoperative complications. Additionally, the MMA group had more patients managed postoperatively for valve or combination CABG plus valve surgery (*p* < 0.0001).

**Table 2:** Descriptive statistics of demographics and confounders vs. pain treatment

Variable	Multimodal (MMA)	Opioid-based	*p* value ^*a*^
N = 762	n = 383	n = 379	
Age, year, median (IQR)	64 (57–71)	66 (59–72)	0.095
Sex, female, N (%)	115 (30%)	78 (20.6%)	0.0035
BMI, kg/m^2^	29.2 (26.1–33.3)	27.7 (25.0–31.7)	0.0006
Race/Ethnicity, N (Col.%)			
Caucasian	227 (59%)	260 (69%)	0.0017 ^b^
Other	156 (41%)	119 (31%)	
African American	124 (32%)	54 (14%)	
Hispanic	6 (1.6%)	10 (2.6%)	
Asian	16 (4.2%)	39 (10%)	
Other/unknown	10 (2.6%)	3 (0.8%)	
Caucasian and Asian	243 (63.5%)	299 (79%)	
Other	140 (36.5%)	80 (21%)	< 0.0001 ^b^
STS risk-adjusted outcomes			
Prolonged postop ventilation	0.063 (0.038–0.10)	0.047 (0.030–0.077)	< 0.0001
Operative Morbidity/mortality	0.101 (0.065–0.16)	0.076 (0.053–0.12)	< 0.0001
Operative mortality	0.012 (0.0064–0.022)	0.0094 (0.0055–0.017)	0.0008
Surgery type, N (%)			< 0.0001
CABG	261 (68%)	318 (84%)	
Valve	81 (21%)	36 (9.5%)	
Both	41 (11%)	25 (6.6%)	
Psychotropics^c^			0.048
No	332 (87%)	308 (81%)	
Yes	51 (13%)	71 (19%)	
# of adjuncts, N (Col. %)^d^			< 0.0001
0	1 (0.3%)	1 (0.3%)	
1	13 (3.4%)	311 (82%)	
2	44 (11.5%)	60 (16%)	
3	296 (77%)	7 (1.8%)	
4	29 (7.6%)	0 (0.0%)	

*BMI* Body Mass Index, *CABG* Coronary Artery Bypass Grafting Surgery, *STS* Society of Thoracic Surgeons

^a^Mann–Whitney U Test, nonparametric equivalent to the t-test for continuous confounders. Fisher Exact Test for 2 × 2 tables or Chi-Square Test for n × 2 tables for categorical confounders

^b^*p* = 0.0017 with Asians grouped with “Other”; *p* < 0.0001 with Asians grouped with Caucasian

^c^Psychotropics: selective serotonin reuptake inhibitor, serotonin-norepinephrine reuptake inhibitor, tricyclic antidepressants, or benzodiazepines

^d^APAP, lidocaine, gabapentinoid or ketorolac. 0–1 adjuncts combined for analysis purposes

There were slightly more patients in the opioid-based group that received concomitant psychotropic medications (*p* = 0.048), e.g., selective serotonin reuptake inhibitors, serotonin-norepinephrine reuptake inhibitors, tricyclic antidepressants, or benzodiazepines. Per differences in the analgesia pathways, 96% in the MMA group received ≥ 2 adjuncts versus 18% in the opioid-based group. Except for four individuals, all patients received APAP.

#### Correlation of confounders

The correlation between PPV, OMM, and OM was very high (r = 0.88–0.97, *p* < 0.0001) thus, only PPV was used for analysis. Patients with higher PPV were older, had higher BMI, were more likely female, were classified as non-white, received > 1 adjunct, and underwent both CABG and valve surgery. All but seven patients (90%) who had CABG plus valve surgery had PPV > 8%, a cut point established for use in later analysis. Regarding race, whites were significantly more likely to be male and receive more psychotropics (Additional file 21).

### Primary outcome: opioid use in MME 72-h post-surgery

#### Unadjusted for fentanyl use

Older patients and females had significantly lower MME (*p* < 0.0001 and *p* = 0.0033, respectively), while patients receiving psychotropics and those with an increased BMI had higher MME use (*p* < 0.0001 and *p* = 0.0003, respectively). The MMA group received more MME than the opioid-based group (Est. 0.22, *p* = 0.0009) (Table 3). However, when stratified into three groups (i.e., < 100 mg, 100–200 mg, > 200 mg), the MMA group was not a significant predictor of the amount of MME consumption (Additional file 21).

**Table 3:** General linear model for MME outcome with and without adjusting for fentanyl use

Outcome	Predictor	Comparison/reference	Estimate (95% CI)	*p* value
Ln MME	Age	–	− 0.029 (− 0.035, − 0.024)	< 0.0001
	Psychotropics^a^	Yes/no	0.40 (0.22, 0.57)	< 0.0001
	BMI	–	0.021 (0.0095, 0.032)	0.0003
	Sex	Female/male	− 0.22 (− 0.36, − 0.07)	0.0033
	Treatment	MMA/opioid-based	0.22 (0.089, 0.34)	0.0009
Ln MME (Fentanyl Adjusted)	Age	–	− 0.031 (− 0.036, − 0.027)	< 0.0001
	Sex	Female/male	− 0.27 (− 0.38, − 0.16)	< 0.0001
	Psychotropics ^a^	Yes/no	0.23 (0.095, 0.36)	0.0007
	BMI	–	0.013 (0.095, 0.36)	0.0031

*MME* Morphine Milligram Equivalents, *MMA* Multimodal analgesia pathway group, *Ln* Natural log, *CI* Confidence Interval

^a^Psychotropics: selective serotonin reuptake inhibitor, serotonin-norepinephrine reuptake inhibitor, tricyclic antidepressants, or benzodiazepines

**Fig. 2:**
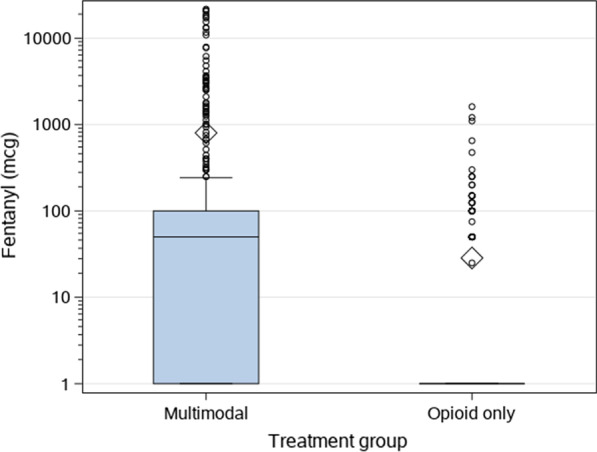
Fentanyl use by treatment group. These boxplots show significantly more liberal use of fentanyl in the MMA group, which accounts for all outlier MME > 500 mg seen in the data. This drives the significantly higher use of MME when MME is treated as a continuous, log-transformed outcome. The rectangles of the boxplot reflect the Q1, median, and Q3 of the data; the diamond is the mean, while circles beyond the whiskers are outliers. For purposes of graphing on a log scale, patients on no fentanyl were marked at 1 mcg. Max value in the MMA group: 21,689 mcg, mean 800 mcg, median 50 mcg. Max in the opioid group: 1610 mcg, mean 28 mcg, median 0 mcg. For conversion, 1000 mcg fentanyl equals 300 mg MME

**Fig. 3:**
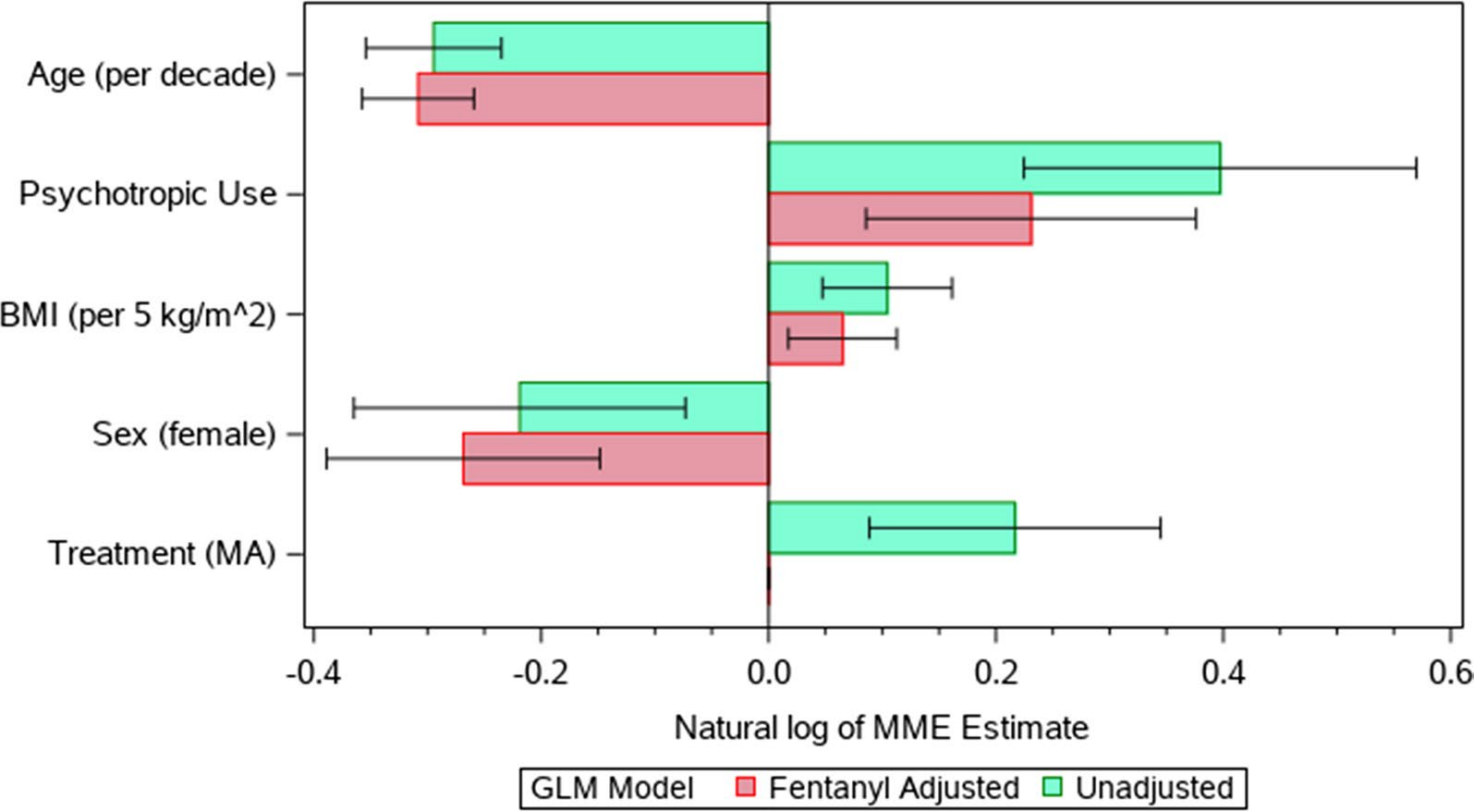
Significant predictors of MME. Comparison of the GLM of MME use, mean estimates with 95% CIs, without and with adjusting for fentanyl protocol differences. Values in parentheses show the comparison group, not the reference. After adjusting for the fentanyl outliers, Treatment Group is no longer a significant predictor of MME. The other predictors remain similar despite the adjustments. Without fentanyl use, there is no significant difference in MME between the MMA and standard opioid groups, which proves that differences in treatment are an artifact of differences in fentanyl use at the different institutions and not differences in the basic protocol

*Adjusted for Fentanyl Use*. There were 41 patients (10.7%) in the MMA group and two patients (0.5%) in the opioid-based group that received ≥ 1000 mcg of IV fentanyl and had a concomitant ≥ 500 mg MME. The MMA group had a median of 50 mcg IV fentanyl (IQR 0–100) and a maximum of 21,689 mcg, while the opioid-based group had a median 0 (IQR 0–0) and a maximum of 1610 mcg IV fentanyl. In the MMA group, the maximum MME consumption was 6527 mg within the 72-h postoperative period, while it was 503 mg in the opioid-based group (Fig. 2). Fentanyl use alone accounted for the total 43 outliers with ≥ 500 mg MME seen in the data. To adjust for these fentanyl outliers, a GLM was run for patients in the opioid-based group only. We replaced MME values > 500 mg with the predicted values of the opioid-based group model to reduce bias that might otherwise occur from simply removing these patients. After adjusting for fentanyl outliers, there is no significant difference in MME between the 2 analgesia groups (Fig. 3).

### Secondary outcomes

#### Mobility within 1 day post-surgery

After adjusting for PPV, patients in the MMA group were more likely to have documented mobility within one-day post-surgery (95% CI 0.31–0.63). Patients with higher PPV were less likely to have documented mobility (*p* < 0.0001) (Table 4).

**Table 4:** Final Multiple Predictor Model for Ambulation, ICU, BM and RASS Outcomes

Outcome	Predictor	Comparison/reference	Odds ratio (95% CI)	*p* value ^a^
Ambulation, Ref: Yes	Ln PPV Treatment	– MMA/Opioid	2.12 (1.61, 2.92) 0.44 (0.31, 0.63)	< 0.0001 < 0.0001
	–	ICU ≤ 60, BM 0–3, RASS 0	Reference group	NA
ICU LOS/BM/RASS	Ln PPV	ICU > 60, BM 0–3, RASS 1–2	2.5 (1.4, 4.3)	0.0009
		ICU > 60, BM 4–9, RASS 0	2.2 (1.2, 3.9)	0.0079
		ICU > 60, BM 4–9, RASS 1–2	2.6 (1.6, 4.4)	0.0002
	Treatment (MM)	ICU ≤ 60, BM 4–9, RASS 1–2	3.8 (1.6, 9.0)	0.0019
		ICU > 60, BM 4–9, RASS 0	2.8 (1.3, 6.2)	0.010
		ICU > 60, BM 4–9, RASS 1–2	3.6 (1.8, 7.2)	0.0004
ICU LOS (≤ 60, > 60)	Ln PPV	–	2.71 (2.14, 3.42)	< 0.0001
	Race/Ethnicity	Other/White-Asian	0.50 (0.35, 0.72)	0.0002
	Psychotropics^b^	Yes/No	2.06 (1.34, 3.18)	0001
	# Adjuncts	2–4/0–1	1.5 (1.10, 2.10)	0.011
BM (0–3 vs 4–9 days)	Ln PPV	–	1.49 (1.11, 1.99)	0.0073
	Treatment	MMA/Opioid	1.93 (1.30, 2.87)	0.0011
RASS (0 vs. 1–2 days)	Ln PPV	–	1.49 (1.14, 1.94)	0.0003
	Treatment	MMA/Opioid	1.62 (1.15, 2.29)	0.0071

*MMA* multimodal analgesia group, *Ln* natural log, *PPV* risk-adjusted Postoperative Prolonged Ventilation, *BM* time to first bowel movement post-surgery, *RASS*: Time to first 0 score post-surgery on the Richmond Agitation-Sedation Scale, CI: Confidence Interval

^a^Only significant comparisons with *p* < 0.017 are listed for ICU/BM/RASS

^b^Psychotropics: selective serotonin reuptake inhibitor, serotonin-norepinephrine reuptake inhibitor, tricyclic antidepressants, or benzodiazepines

#### ICU LOS, time to first BM, and time to first zero RASS post-surgery (combined outcomes)

Ln PPV and the analgesia groups were significant predictors. Results showed that the combined outcome was statistically significant (*p* < 0.0001, globally), allowing each variable to be analyzed separately (Table 4).

*ICU LOS.* Analysis of ICU LOS in two groups (≤ 60-h, > 60-h) and ICU LOS as a continuous, log-transformed variable in the GLM provided similar results. In both methods (i.e., dichotomous and continuous analysis), Ln PPV, race/ethnicity, and psychotropics were significant predictors of ICU LOS. The analgesia arms did not appear in either analysis and were therefore not a predictor for ICU LOS. The number of adjuncts given was a significant predictor when ICU LOS was dichotomized but not with ICU LOS as a continuous, log-transformed outcome.

#### ICU LOS and race

Patients in either analgesia group who classified as African American or other race were significantly more likely than Caucasians or Asians to have ≤ 60-h ICU LOS if PPV was < 8%; 75% of African Americans/other race versus 62% of Caucasians/Asians (*p* = 0.0077, Fisher Exact Test) (Additional file 21).

#### Time to first BM post-surgery and time to first zero RASS post-surgery

After adjusting for PPV, patients in the MMA group were 1.9 times more likely to have first BM at 4–9 days (*p* = 0.0011) and 1.6 times more likely to achieve first zero RASS at 1–2 days (*p* = 0.0071).

## Discussion

In this retrospective observational study within a single healthcare system, the authors did not observe an association between the use of a postoperative MMA pathway and the reduction in the primary outcome of opioid consumption (i.e., MME) after cardiac surgery. Similarly, no association was found between MMA and reduction in the secondary outcomes of ICU LOS, time to first BM, and time to first zero RASS in an analysis risk-adjusting for PPV. Interestingly, patients in the MMA group were more likely to have documented mobility within one-day post-surgery.

The impact of MMA in reducing opioid consumption, decreasing adverse effects, and improving postoperative recovery is well documented in the literature [1, 9, 16]. A variety of MMA protocols with or without the addition of regional anesthesia techniques have been studied. Contrary to our findings, a randomized trial in cardiac surgery patients reported lower pain scores on the numeric rating scale in the MMA group on postoperative days 0–3 [4]. Differing from this work, the addition of regional anesthesia techniques and a standardized anesthesia protocol might explain the reduction in total MME utilization, higher number of extubations in the operating room, and reduction in total intubation times [17]. Improved pain scores at 6-h post-surgery and decreased opioid consumption on postoperative day one were demonstrated when intraoperative fentanyl was substituted with a combination of methadone, ketamine, dexmedetomidine, and ketorolac [18].

The use of APAP and gabapentinoids are effective and commonly used in cardiac surgery patients [1, 4, 8]. Anwar et al. [19] reported that patients receiving pregabalin 150 mg, preoperatively, followed by 300 mg for 14 days post-surgery had significant reductions in surgical site pain, morphine consumption, and persistent pain at both three and six months. In comparison, the MMA protocol at our institution does not include pre-emptive pregabalin and lower doses of pregabalin are only prescribed for four days post-surgery. With the availability of a standardized dose for neuropathic pain and a lower incidence of drowsiness, pregabalin 150 mg in divided doses was selected as the preferred gabapentinoid for this MMA regimen unless the patient was previously on a stable dose of gabapentin as an outpatient [20]. Since implementing this protocol, a recently published meta-analysis refutes previous claims of analgesic efficacy and now suggests that gabapentinoids pose a higher risk of adverse drug reactions (e.g., dizziness, visual disturbance) with no clinically significant benefit [21].

As an adjunct after cardiac surgery, 4 gm of IV APAP administered during the first 24-h reduced incisional pain [22–25]. At the two MMA facilities in this study, 650 mg of oral APAP every 6-h for three days was pre-selected for all patients unless contraindicated. At the time of protocol development, standardized use of IV APAP was cost-prohibitive and thus, not included in the regimen. However, studies comparing IV APAP with either oral or rectal APAP have determined that IV administration did not improve pain scores [25, 26].

While topical lidocaine and lidocaine infusions have proven beneficial in non-cardiac surgical patients[27], there are conflicting reports in cardiac surgery patients. Some studies describe positive effects on analgesia, whereas others show no impact. Applying topical lidocaine patches at both chest tube and sternotomy sites resulted in significantly lower amounts of fentanyl delivered via patient-controlled analgesia (PCA) [28]. However, in a study of 39 patients after robotic cardiac valve surgery, 5% lidocaine transdermal patches with PCA were ineffective at reducing acute or persistent pain [29]. Similar findings were also noted in patients receiving lidocaine in addition to other opioid and non-opioid analgesics following a thoracotomy and sternotomy [30].

Hypothetically, a MMA regimen consisting of scheduled APAP, gabapentinoid, and transdermal lidocaine should provide synergistic analgesia and thus, minimize opioid consumption. However, in this study, the prescription of postoperative MMA did not reduce opioid utilization. Studies suggest that the nociceptive pathway is inundated with noxious stimuli from surgical trauma leading to severe pain secondary to central sensitization and hyperalgesia [31, 32]. For patients placed on cardiopulmonary bypass, an even higher degree of pain is often experienced due to inflammation-mediated by cardiopulmonary bypass [31, 32]. Furthermore, using high-dose, short-acting opioids as a component of perioperative anesthesia has been associated with higher pain intensity, hyperalgesia, and increased use of postoperative opioids [2, 26, 33]. Thus, preventive analgesia may be more effective at reducing acute postoperative pain and subsequent opioid consumption [1, 4, 16, 17]. In addition to preventive MMA, the use of long-acting opioids, opioid-free anesthesia, regional anesthesia techniques, and/or alternative anesthetics such as dexmedetomidine and ketamine have also been proposed as mitigation strategies [18, 26, 33].

## Limitations

Although this study was conducted within a single healthcare system, the analgesia groups evaluated were located at geographically separate institutions. Based on risk-adjusted outcomes for PPV, OMM, and OM, patients in the MMA group were at significantly higher risks of postoperative complications. This may be attributed to the higher percentage of uninsured patients serviced at the two hospitals utilizing MMA compared to the hospital using opioid-based regimens, 14% versus 2% [34].

While we excluded patients with documented chronic pain, we did not further exclude patients with preoperative opioid consumption. The rate of preoperative opioid consumption in cardiac surgery patients has been reported at 10.8% [35]. Preoperative opioid use is a strong predictor of long-term postoperative opioid use [36] and might also influence opioid use in the acute postoperative phase.

Another limiting factor common with other retrospective studies was the inability to control confounders not included in the model. We were rigorous in identifying major categories of these confounders, including baseline opioid use and patient comorbidities via the STS risk-adjusted outcome rates for PPV, OM, and OMM. Additionally, it is worth noting that slightly more patients in the opioid-based group received concomitant psychotropic medications than those in the multimodal group. While the authors were unable to validate the degree to which this concomitant therapy impacted the results of this study, the potential of these agents to directly or indirectly alter responses to painful stimuli should be considered [37, 38]. Incidentally, the analgesia groups were not a predictor for ICU LOS in the models however, they can be impacted (i.e., shortened, prolonged) by situations related to patient flow, hospital policy, staffing, or other unknown factors.

Further, differences in prescribing practices by rotating ICU clinicians can contribute to the observed dissimilarities in opioid usage. For instance, providers utilizing the opioid-based pathway still had the option to order non-opioid analgesics such as IV ketorolac, topical lidocaine, and/or oral pregabalin to optimize pain management; this, in addition to the standardized order for as needed oral APAP. In this cohort, only 18% of patients in the opioid-based group received ≥ 2 adjuncts however, any use of non-opioid adjuncts may have contributed to reduced MME. Additionally, with the steady onboarding of new ICU nurses, the authors recognize that varying degrees of nursing experience may account for differences in pain assessment and management (e.g., IV versus oral administration, timing of doses). The authors also realize that the potential for implicit bias to negatively impact pain management cannot be discounted at this time. Analgosedation is typically managed with opioid infusions titrated via a nursing-driven protocol for mechanically ventilated patients. Without conducting an audit, the authors could not confirm that nurses titrated these infusions consistently. Since this protocol was implemented, the institution has revised the titration parameters for analgosedation to help mitigate variations in practice. Additionally, the inability to control for the amount of analgosedation needed to achieve targeted RASS goals for mechanically ventilated patients is another limitation as MME utilization becomes skewed. In retrospect, the initial utilization of this MMA pathway would have been more appropriate for pre-identified patients deemed eligible for ERAS or “fast-track surgery” protocols [10]. Finally, it would have been valuable to assess the patients’ pain experience using the numerical pain rating scale (NPRS) in this analysis in addition to using RASS. However, the NPRS would need to be appropriately administered and consistently documented at standard intervals to have the greatest value in providing additional data.

Studies demonstrating reduced MME consumption secondary to MMA consistently outlined a standardized perioperative MMA pathway, whereas this study only evaluated a postoperative MMA pathway. The lack of standardized preoperative and intraoperative analgesia pathways at our institution is another limitation of this study. Future plans at this institution include the development of a MMA pathway that incorporates standardized management in both the preoperative and intraoperative periods.

Non-steroidal anti-inflammatory drugs (NSAIDs) have proven effective in reducing opioid consumption and opioid-related complications. However, an increase in cardiovascular thrombotic events mediated by cyclooxygenase-2 selective inhibitors was previously documented in the literature. This resulted in the issuance of a black box warning suggesting that perioperative use of NSAIDs in CABG surgery was contraindicated. However, several meta-analyses have failed to demonstrate similar findings (i.e., cardiovascular events) with short-term administration of nonselective NSAIDs in select patients undergoing cardiac surgery [39–42]. Given that this postoperative MMA pathway was intended for global use within the two CTS ICUs, NSAIDs were not prescribed for routine administration.

### Next steps

Recently, a multidisciplinary cardiac analgesia optimization team at one of the three CTS ICUs was formed to develop and evaluate the efficacy of a robust perioperative MMA pathway. After a preliminary meeting, this team aims to establish a routine preoperative MMA regimen and standardize intraoperative practices to include both MMA administration and regional anesthetic blocks. As it relates to the existing postoperative MMA pathway, the following adjustments will also be considered:

- Current literature suggests maximal analgesia was experienced within the 24-h post-surgery period when a total of 4 gm IV APAP were administered [22, 23]. To remain within the constructs of the hospital formulary, APAP liquid should be substituted to allow for enteral doses of 4 gm APAP within 24-h then reduced to 650 mg every 6-h for an additional three days.
- Although Verrett et al. found no clinically significant difference in opioid consumption with the use of gabapentinoids, the meta-analysis did not provide details on the concomitant therapy patients were receiving [27]. As such, the potential role for gabapentinoids may require modification (e.g., timing of administration, duration of therapy) in this MMA protocol instead of being eliminated.
- Incorporation of NSAIDs (e.g., ketorolac or naproxen) for short-term administration in patients with low risks (i.e., normal baseline renal function, less likely to have prolonged postoperative ventilation) [39–42]. One potential option includes 15 mg IV ketorolac every 6-h for the first 24-h, followed by naproxen 500 mg twice daily for three days.

This quality initiative will be trialed in select CTS patients before considering system-wide expansion.

Preoperative anxiety, catastrophizing and decreased response to conditioned pain modulation have been identified as risk factors for patients likely to experience persistent, uncontrolled postoperative pain. Patients receiving preoperative education have less anxiety, lower pain scores, reduced hospital LOS, and a faster return to their baseline [9, 19]. Thus, it is recommended that patients receive preoperative counseling to help establish appropriate expectations for postoperative pain and proper management [9, 19, 26, 32]. While some degree of preoperative education is provided, the authors recognize that the development of robust, standardized education designed to assist the patient in establishing realistic postoperative pain expectations might further enhance outcomes.

## Conclusion

The use of a MMA pathway did not reduce total opioid consumption during the immediate postoperative period after a conventional CABG and/or valve surgery via median sternotomy. Patients in the MMA group were more likely to have documented mobility within one-day post-surgery; however, they were also more likely to have a longer time until first BM and first zero RASS. While the findings of this study do not support the initial hypothesis, the authors believe that the development and assessment of a robust standardized perioperative (as opposed to postoperative) MMA approach may be warranted. Although the analgesia groups were not a significant predictor for ICU LOS, potential racial disparities were an unanticipated finding that requires further investigation.

## Supplementary Information

**Additional file 1**. Because of the high correlation between PPV, OMM, and OM (r = 0.88-0.97), only PPV was used in models. PPV was correlated with both Age and BMI. Age was negatively correlated with BMI.

**Additional file 2**. Statistical Tests: Mann-Whitney U Test for race, sex, and # of adjuncts vs. continuous variables. Kruskal-Wallis Test for Surgery Type vs. continuous variables. Chi-Square Test by n x m categorical variables. Fisher Exact Test for 2 x 2 categorical variables. Abbreviations: PPV-Predicted Prolonged Ventilation; BMI-Body Mass Index; Psych. - Psychotropic drugs.

**Additional file 3**. Abbreviations: MME- Morphine Milligram Equivalents, ICU - Intensive Care Unit, BM - Time (days) to first bowel movement, Extub. - Time (days) to Extubation, Vent. - Time (hr) on ventilator, RASS- Time (days) to first zero score on Richmond-Agitation-Sedation Scale, NS- Not statistically significant (p > 0.017).

**Additional file 4**. ^a ^Mann-Whitney U Test for continuous outcomes, Fisher Exact Test for 2 x 2 categorical outcomes.  Abbreviations: IQR: Interquartile Range; MME: Morphine Milligram Equivalents; CAM-ICU: Confusion Assessment Method; ICU: Intensive Care Unit; RASS: Richmond-Agitation-Sedation Scale; BM: Bowel Movement.

**Additional file 5**. Multinomial Logistic Regression of MME.

**Additional file 6**. ^a^ Fisher Exact Test.  Abbreviations: ICU: Intensive Care Unit; PPV: Postoperative Prolonged Ventilation; Af. Amer.: African American.

**Additional file 7**. ^a^Fisher Exact Test of treatment type versus time on the ventilator (cut at 11 hours) within each surgery type.  Abbreviations: CABG: Coronary Artery Bypass surgery.

**Additional file 8**. ^a^ Odds ratio for binary logistic regression, > 6-h vs ≤ 6-h ventilator time. ^b^ Note that while this is significant at α = 0.05 threshold, it is not at a reduced threshold of 0.017.  Abbreviations: NA: Not applicable; GLM: General Linear Model; Ln: Natural log; PPV: Risk-adjusted Postoperative Prolonged Ventilation; CABG: Coronary Artery Bypass Graft surgery; NS: Not significant; MM: Multimodal treatment group.

**Additional file 9**. MME vs Age by Treatment Group. When MME is divided into 3 groups split at 100 and 200 mg, the treatment group is not significantly different. However, when MME is treated as continuous, it is significant because of outlier levels of MME. This is driven by differences in fentanyl use at the different institutions. All outliers > 700 mg are from fentanyl use.

**Additional file 10**.  Time in ICU versus Postoperative Prolonged Ventilation by Race. White/Asian vs. African Amer. and Other. Results show that with a PPV less than 8%, African Americans and other races were significantly more likely to have an ICU time below 60-h, 75% vs 62%. 

**Additional file 11**. Scatterplot of Time on Ventilator vs Time in ICU by Treatment Group. Time on Ventilators was significantly correlated with Time in the ICU (p < 0.0001, r = 0.42, Spearman Rank Correlation). Because of this high correlation, only Time in ICU was used as an outcome. Breaks in ICU time at 36-h, 60-h, and 84-h (i.e., 1.5, 2.5, and 3.5 days) reflect lower discharge rates during the nighttime. Time in the ICU was dichotomized at 60-h for purposes of the regression analysis. Patients in the multimodal group had significantly longer times on the ventilator (p = 0.0061, Mann-Whitney U Test) and borderline longer time in the ICU (p = 0.058, Mann-Whitney U Test); however, these differences disappear from both regression models after adjusting for PPV and psychotropic use. For purposes of graphing on a log scale, 3 patients with no time on the ventilator were marked at 0.1-h.

**Additional file 12**. Histogram of Ventilation Hours by Treatment. Time on the ventilator is significantly higher in the multimodal analgesia group than the opioid only group (p = 0.0061, Mann-Whitney U Test). The histogram of the natural log of ventilator hours shows a positive skew in the opioid group. However, it is bimodal in the multimodal group, with a dip at 2.375, corresponding to 11-h, and a peak at 3.0, corresponding to 20-h. These unexpected results cannot be explained alone by the greater proportion of patients who had both CABG and valve surgeries (11% vs 6.6%). Note that for purposes of taking a natural log transformation, 3 patients without any time on the ventilator were marked as having spent 0.1-h, which translates to -2.3 on a log scale.

**Additional file 13**. Histogram of Ln PPV by Treatment Group. The multimodal analgesia group was significantly more likely to have a higher risk-adjusted postoperative prolonged ventilation (PPV, p < 0.0001, Mann-Whitney U Test). Even after log transformation, the data are slightly positively skewed. However, the data were treated as if they were normally distributed for regression analysis purposes. 

**Additional file 14**. Scatterplot of Body Mass Index (BMI) vs Age by Treatment Group. Body mass index is significantly negatively correlated with age (p = 0.003, r = −0.13, Spearman Rank Correlation). Linear regression trend lines were not drawn because of outlier patients especially in the multimodal group with many younger, normal weight patients. This would have violated assumptions of the test. 

**Additional file 15**. Scatterplot Time to Extubation vs Time on Ventilator by Treatment Group. Time to Extubation was highly correlated with Time on Ventilator (p < 0.0001, r = 0.73, Spearman Rank Correlation). Because of the high correlation, because Time to Extubation was only available for Coronary Artery Bypass Graft surgery patients, and because it was measured in days instead of hours, this variable was dropped as an outcome. Three patients with no time on the ventilator were omitted for purposes of graphing on a log scale.

**Additional file 16**. Scatterplot Time to First Bowel Movement (BM) vs Time in ICU. Time to first bowel movement (BM) was significantly correlated with time in ICU (p < 0.0001, r = 0.24, Spearman Rank Correlation). Data have been jittered about the BM axis to separate data points.

**Additional file 17**.  Scatterplot Time to First Zero RASS vs Time in ICU by Treatment. Results show a significant association of time to first zero score on the Richmond-Agitation-Sedation Scale (RASS) and Time in ICU (p = 0.0003, r = 0.15, Spearman Rank Correlation). Data have been jittered about the RASS axis to facilitate ease of viewing individual data points.

**Additional file 18**. Scatterplot Time in ICU vs PPV by Surgery Type. Time in ICU was significantly correlated with Prolonged Postoperative Ventilation (PPV, p < 0.0001, r = 0.37, Spearman Rank Correlation). Both Time in ICU and PPV were significantly associated with surgery type (p < 0.0001, Kruskal Wallis Test). Ninety percent of patients who had "both" Coronary Artery Bypass Graft (CABG) and heart valve surgeries (green) had a PPV over 8% (grey reference line).

**Additional file 19**. MME vs PPV by Treatment Group. Morphine Milligram Equivalents (MME) was significantly negatively associated with Prolonged Postoperative Ventilation (p < 0.0001, r = −0.16, Spearman Rank Correlation). Patients with MME > 700 mg in the MMA group were all given fentanyl. For purposes of graphing on a log scale, patients with no MME were marked at 1 mg.

**Additional file 20**. MME vs Time on Ventilator by Treatment Group. Morphine Milligram Equivalents (MME) was not significantly associated with Time on Ventilator (p = 0.45), Spearman Rank Correlation) nor with Time in ICU (data not show, p = 0.17). However, patients with MME > 700 mg in the MMA group, who were all given fentanyl, all had longer ventilator times of > 10-h. But patients with long time on the ventilator did not all have outlier levels of MME. For purposes of graphing on a log scale, patients with no MME were marked 1 mg and patients with no ventilator time were marked at 0.1-h.

**Additional file 21**. Summary of Supplemental Data.

## Data Availability

Datasets are available from the corresponding author upon reasonable request.

## Abbreviations

APAP: Acetaminophen
APS: American Pain Society
BM: Bowel movement
BMI: Body mass index
CABG: Coronary artery bypass grafting
CTS ICU: Cardiothoracic surgery intensive care unit
ERAS: Enhanced Recovery After Surgery
GLM: General Linear Model
Ln PPV: Natural log of prolonged postoperative ventilation
LOS: Length of stay
MMA: Multimodal analgesia
MME: Morphine milligram equivalents
OM: Operative mortality
OMM: Operative major morbidity or mortality composite
PPV: Postoperative prolonged ventilation
RASS: Richmond Agitation-Sedation Scale
STS: Society of Thoracic Surgeons
VAS: Visual Analog Scale
