# Correction: The impact of a postoperative multimodal analgesia pathway on opioid use and outcomes after cardiothoracic surgery

**DOI:** 10.1186/s13019-023-02149-w

**Published:** 2023-01-23

**Authors:** Ceressa T. Ward, Vanessa Moll, David W. Boorman, Lijo Ooroth, Robert F. Groff, Trent D. Gillingham, Laura Pyronneau, Amit Prabhakar

**Affiliations:** 1 Convergent Genomics, 425 Eccles Avenue, South San Francisco, CA 94080 USA; 2 Department of Anesthesiology, Emory University School of Medicine, Atlanta, GA USA; 3 Mercer University College of Pharmacy, Atlanta, GA USA; 4 Office of Quality, Emory Healthcare, Atlanta, GA USA; 5 CVS Pharmacy, Douglasville, GA USA; 6 Potrero Medical, Hayward, CA USA

**Correction to: Journal of Cardiothoracic Surgery (2022) 17:342** https://doi.org/10.1186/s13019-022-02067-3

The original online version of this article was revised: Vanessa Moll treated as a equally contributed author now this has been corrected.

