# Supplementary material for: The impact of a postoperative multimodal analgesia pathway on opioid use and outcomes after cardiothoracic surgery
Source: J Cardiothorac Surg. 2022 Dec 30;17:342. doi: 10.1186/s13019-022-02067-3 (PMC9801617; doi:10.1186/s13019-022-02067-3)
Supplement: Supplementary file 1 — Additional file 1. Because of the high correlation between PPV, OMM, and OM (r = 0.88-0.97), only PPV was used in models. PPV was correlated with both Age and BMI. Age was negatively correlated with BMI. [file 13019_2022_2067_MOESM1_ESM.docx]

**Table S1: Spearman Rank Correlation of Continuous Confounders**

| Variables | Age | BMI | PPV | OMM | OM |
| --- | --- | --- | --- | --- | --- |
| Age | r | -0.13 | 0.29 | 0.30 | 0.48 |
|  | p-value | 0.0003 | <.0001 | <.0001 | <.0001 |
| BMI | -0.13 | r | 0.11 | 0.078 | NS |
|  | 0.0003 | p-value | 0.0019 | 0.033 | 0.12 |
| PPV | 0.29 | 0.11 | r | 0.97 | 0.88 |
|  | <.0001 | 0.0019 | p-value | <.0001 | <.0001 |
| OMM | 0.30 | 0.078 | 0.97 | r | 0.92 |
|  | <.0001 | 0.033 | <.0001 | p-value | <.0001 |
| OM | 0.48 | NS | 0.88 | 0.92 | r |
|  | <.0001 | 0.12 | <.0001 | <.0001 | p-value |
| *Abbreviations: BMI-Body Mass Index; ICU-Intensive Care Unit; Vent Hr-Hours on a ventilator; PPV-Predicted Prolonged Ventilation; OMM-Operative Morbidity and Mortality Composite; OM-Operative Mortality; NS-Not statistically significant* | | | | | |
