# Supplementary material for: The impact of a postoperative multimodal analgesia pathway on opioid use and outcomes after cardiothoracic surgery
Source: J Cardiothorac Surg. 2022 Dec 30;17:342. doi: 10.1186/s13019-022-02067-3 (PMC9801617; doi:10.1186/s13019-022-02067-3)
Supplement: Supplementary file 2 — Additional file 2. Statistical Tests: Mann-Whitney U Test for race, sex, and # of adjuncts vs. continuous variables. Kruskal-Wallis Test for Surgery Type vs. continuous variables. Chi-Square Test by n x m categorical variables. Fisher Exact Test for 2 x 2 categorical variables. Abbreviations: PPV-Predicted Prolonged Ventilation; BMI-Body Mass Index; Psych. - Psychotropic drugs. [file 13019_2022_2067_MOESM2_ESM.docx]

**Table S2: Association of Categorical Confounders**

| Confounder | Race/Ethnicity | | Sex | | Surgery Type | | | Number of Adjuncts | |
| --- | --- | --- | --- | --- | --- | --- | --- | --- | --- |
|  | **p-value** | **Direction** | **p-value** | **Direction** | **p-value** | **Direction** | | **p-value** | **Direction** |
|  |  | Nonwhites have |  | Females have |  | Valves have | Both have |  | 2-4 adjuncts have |
| PPV | <0.0001 | 🡩 PPV | <0.0001 | 🡩 PPV | <0.0001 | -- | 🡩 PPV | <0.0001 | 🡩 PPV |
| Age | <0.0001 | 🡫 Age | 0.27 | -- | 0.0007 | 🡫 Age | 🡩 Age | 0.11 | -- |
| BMI | 0.043 | 🡫 BMI | 0.53 | -- | 0.18 | -- | -- | 0.024 | 🡩 BMI |
| Race | NA | -- | 0.0005 | 🡩 Nonwhite | 0.35 | -- | -- | 0.080 | -- |
| Sex | 0.0005 | 🡩 Female | NA | -- | 0.0032 | 🡩 Female | -- | 0.015 | 🡩 Female |
| Surg. Type | 0.35 | -- | 0.0032 | 🡩 Valve | NA | -- | -- | 0.0002 | 🡩 Valve |
| # Adjuncts | 0.080 | -- | 0.015 | 🡩 2-4 adj | 0.0002 | 🡩 2-4 adj | -- | NA | -- |
| Psych. | <0.0001 | 🡫 Psych. | 0.82 | -- | <0.0001 | 🡩 Psych. | -- | 0.058 | -- |

Statistical Tests: Mann-Whitney U Test for race, sex, and # of adjuncts vs. continuous variables. Kruskal-Wallis Test for Surgery Type vs. continuous variables. Chi-Square Test by n x m categorical variables. Fisher Exact Test for 2 x 2 categorical variables.

Abbreviations: PPV-Predicted Prolonged Ventilation; BMI-Body Mass Index; Psych.-Psychotropic drugs.
