# Supplementary material for: The impact of a postoperative multimodal analgesia pathway on opioid use and outcomes after cardiothoracic surgery
Source: J Cardiothorac Surg. 2022 Dec 30;17:342. doi: 10.1186/s13019-022-02067-3 (PMC9801617; doi:10.1186/s13019-022-02067-3)
Supplement: Supplementary file 3 — Additional file 3. Abbreviations: MME- Morphine Milligram Equivalents, ICU - Intensive Care Unit, BM - Time (days) to first bowel movement, Extub. - Time (days) to Extubation, Vent. - Time (hr) on ventilator, RASS- Time (days) to first zero score on Richmond-Agitation-Sedation Scale, NS- Not statistically significant (p > 0.017). [file 13019_2022_2067_MOESM3_ESM.docx]

**Table S3: Spearman Rank Correlation (r) of Continuous Outcome Variables**

| Outcome | N | Grouped/Transform | MME | ICU | BM | Extub | Vent | RASS |
| --- | --- | --- | --- | --- | --- | --- | --- | --- |
| MME | 762 | Natural Log of mg | -- | NS | NS | NS | NS | NS |
| ICU | 762 | ≤ 60 hr vs > 60 hr | NS | -- | 0.24 | 0.26 | 0.42 | 0.15 |
| BM | 428 | 0-3 days vs 4-9 days | NS | 0.24 | -- | 0.13 | 0.22 | NS |
| Extubation | 550 | 0, 1, 2+ days | NS | 0.27 | 0.13 | -- | 0.73 | 0.43 |
| Ventilation | 762 | Natural Log of hr | NS | 0.42 | 0.22 | 0.73 | -- | 0.44 |
| RASS | 550 | 0 days vs 1-2 days | NS | 0.15 | NS | 0.43 | 0.44 | -- |

Abbreviations: MME- Morphine Milligram Equivalents, ICU- Intensive Care Unit, BM- Time (days) to first bowel movement, Extub.-Time (days) to Extubation, Vent.- Time (hr) on ventilator, RASS- Time (days) to first zero score on Richmond Agitation Sedation Scale, NS- Not statistically significant (p>0.017)
