# Supplementary material for: The impact of a postoperative multimodal analgesia pathway on opioid use and outcomes after cardiothoracic surgery
Source: J Cardiothorac Surg. 2022 Dec 30;17:342. doi: 10.1186/s13019-022-02067-3 (PMC9801617; doi:10.1186/s13019-022-02067-3)
Supplement: Supplementary file 4 — Additional file 4. a Mann-Whitney U Test for continuous outcomes, Fisher Exact Test for 2 x 2 categorical outcomes. Abbreviations: IQR: Interquartile Range; MME: Morphine Milligram Equivalents; CAM-ICU: Confusion Assessment Method; ICU: Intensive Care Unit; RASS: Richmond-Agitation-Sedation Scale; BM: Bowel Movement. [file 13019_2022_2067_MOESM4_ESM.docx]

**Table S4: Preliminary Analysis of Outcomes by Treatment Group, Unadjusted**

| Outcome, median (IQR) or N (%) | Set | N | Multimodal | Stnd Opioid | p-value^a^ |
| --- | --- | --- | --- | --- | --- |
| MME | 1 | 762 | 152 (92-235) | 145 (86.5-223.5) | 0.23 |
| Ambulation, No, N (%) | 2 | 552 | 96 (39%) | 165 (54%) | 0.0006 |
| CAM-ICU Delirium, Yes, N (%) | Omitted | 552 | 4 (1.6%) | 6 (2.0%) | 0.62 |
| Time to Extubation (days) | Omitted | 550 | 0 (0-1) | 0 (0-1) | 0.0077 |
| Time on Ventilator (hours) | Omitted | 762 | 6.6 (3.9-15.7) | 5.5 (4.1-9.4) | 0.0061 |
| Time in ICU (hours) | 3 | 762 | 56 (31-92) | 50 (30-77) | 0.058 |
| Time to first bowel movement (days) | 3 | 428 | 4 (3-4) | 3 (3-4) | 0.0004 |
| Time to first 0 RASS score (days) | 3 | 550 | 1 (0-1) | 0 (0-1) | 0.0004 |
| Time in ICU > 60 hours, N (%) | 3 | 762 | 184 (48%) | 149 (39%) | 0.016 |
|  |  | 428 | 101 (48%) | 80 (37%) | 0.024 |
| Time to first BM, 4-9 days, N (%) | 3 | 428 | 110 (52%) | 75 (35%) | 0.0003 |
| Time to first 0 RASS score, 1-2 days, N (%) | 3 | 550 | 142 (58%) | 136 (44%) | 0.0015 |
|  |  | 428 | 120 (57%) | 93 (43%) | 0.0037 |

^a^ Mann-Whitney U Test for continuous outcomes, Fisher Exact Test for 2x2 categorical outcomes

Abbreviations: IQR: Interquartile Range; MME: Morphine Milligram Equivalents; CAM-ICU: Confusion Assessment Method; ICU: Intensive Care Unit; RASS: Richmond Agitation Sedation Scale; BM: Bowel Movement
