# Supplementary material for: The impact of a postoperative multimodal analgesia pathway on opioid use and outcomes after cardiothoracic surgery
Source: J Cardiothorac Surg. 2022 Dec 30;17:342. doi: 10.1186/s13019-022-02067-3 (PMC9801617; doi:10.1186/s13019-022-02067-3)
Supplement: Supplementary file 5 — Additional file 5. Multinomial Logistic Regression of MME. [file 13019_2022_2067_MOESM5_ESM.docx]

**Table S5: Multinomial Logistic Regression of MME**

| **Outcome** | **Predictor** | **Comparison / Reference** | **Odds Ratio (95% CI)** | **p-value** |
| --- | --- | --- | --- | --- |
| MME 3 gp:  <100,  100-199,  200+ | Age | MME 100-199/<100 | 0.33 (0.25-0.42) / 10 yr | <0.0001 |
|  |  | MME 200+/<100 | 0.19 (0.15-0.25) / 10 yr | <0.0001 |
|  | Psychotropics (Y) | MME 100-199/<100 | -- | NS |
|  |  | MME 200+/<100 | 3.24 (1.77-5.94) | <0.0001 |
|  | Sex (Female) | MME 100-199/<100 | 0.50 (0.33-0.77) | 0.0013 |
|  |  | MME 200+/<100 | 0.37 (0.22-0.62) | <0.0001 |
