# Supplementary material for: The impact of a postoperative multimodal analgesia pathway on opioid use and outcomes after cardiothoracic surgery
Source: J Cardiothorac Surg. 2022 Dec 30;17:342. doi: 10.1186/s13019-022-02067-3 (PMC9801617; doi:10.1186/s13019-022-02067-3)
Supplement: Supplementary file 6 — Additional file 6. a Fisher Exact Test. Abbreviations: ICU: Intensive Care Unit; PPV: Postoperative Prolonged Ventilation; Af. Amer.: African American. [file 13019_2022_2067_MOESM6_ESM.docx]

**Table S6: ICU Time vs. PPV by Race/Ethnicity**

| N (%) |  | PPV | |
| --- | --- | --- | --- |
| ICU Time | **Race** | **< 8%** | **> 8%** |
| < 60 hr | Caucasian-Asian | 246 (62%) | 45 (31%) |
|  | Af. Amer.-Other | 98 (75%) | 38 (44%) |
| > 60 hr | Caucasian-Asian | 148 (38%) | 100 (69%) |
|  | Af. Amer.-Other | 32 (25%) | 48 (56%) |
| p-value^a^ |  | 0.0077 | 0.0482 |

**^a^** Fisher Exact Test

Abbreviations: ICU: Intensive Care Unit; PPV: Postoperative Prolonged Ventilation; Af. Amer.: African American
