# Supplementary material for: The impact of a postoperative multimodal analgesia pathway on opioid use and outcomes after cardiothoracic surgery
Source: J Cardiothorac Surg. 2022 Dec 30;17:342. doi: 10.1186/s13019-022-02067-3 (PMC9801617; doi:10.1186/s13019-022-02067-3)
Supplement: Supplementary file 7 — Additional file 7. aFisher Exact Test of treatment type versus time on the ventilator (cut at 11 hours) within each surgery type. Abbreviations: CABG: Coronary Artery Bypass surgery. [file 13019_2022_2067_MOESM7_ESM.docx]

**Table S7: Proportion of Patients above 11 Ventilator Hours**

| Treatment Group 🡪 | | Multimodal Analgesia | | Opioid Only | | Total | |  |
| --- | --- | --- | --- | --- | --- | --- | --- | --- |
| Time on Ventilator 🡪 | | **≤ 11 hr** | **> 11 hr** | **≤ 11 hr** | **> 11 hr** | **≤ 11 hr** | **> 11 hr** | **p-value ^a^** |
| Surgery | CABG | 184 (70%) | 77 (30%) | 269 (85%) | 49 (15%) | 453 (78%) | 126 (22%) | <0.0001 |
|  | Valve | 55 (68%) | 26 (32%) | 23 (64%) | 13 (36%) | 78 (67%) | 39 (33%) | 0.68 |
|  | Both | 15 (37%) | 26 (63%) | 14 (56%) | 11 (44%) | 29 (44%) | 37 (56%) | 0.14 |
|  | Total | 254 (66%) | 129 (34%) | 306 (81%) | 73 (19%) | 29 (44%) | 37 (56%) | <0.0001 |

^a^ Fisher Exact Test of treatment type versus time on the ventilator (cut at 11 hours) within each surgery type.

Abbreviations: CABG: Coronary Artery Bypass Graft surgery
