# Supplementary material for: The impact of a postoperative multimodal analgesia pathway on opioid use and outcomes after cardiothoracic surgery
Source: J Cardiothorac Surg. 2022 Dec 30;17:342. doi: 10.1186/s13019-022-02067-3 (PMC9801617; doi:10.1186/s13019-022-02067-3)
Supplement: Supplementary file 8 — Additional file 8. a Odds ratio for binary logistic regression, > 6-h vs ≤ 6-h ventilator time. b Note that while this is significant at α = 0.05 threshold, it is not at a reduced threshold of 0.017. Abbreviations: NA: Not applicable; GLM: General Linear Model; Ln: Natural log; PPV: Risk-adjusted Postoperative Prolonged Ventilation; CABG: Coronary Artery Bypass Graft surgery; NS: Not significant; MM: Multimodal treatment group. [file 13019_2022_2067_MOESM8_ESM.docx]

**Table S8: Regression of Time on Ventilator**

| Outcome | Predictor | Comparison / Reference | Estimate  (95% CI) ^a^ | Odds Ratio or GLM | p-value |
| --- | --- | --- | --- | --- | --- |
| Ln Ventilator Time | Intercept | NA | 3.17 (2.93-3.41) | GLM | <0.0001 |
|  | Ln PPV | NA | 0.44 (0.36-0.52) |  | <0.0001 |
|  | Psychotropics | Yes / No | 0.24 (0.0715-0.41) |  | 0.0052 |
| Ventilator Time > 6 hrs | Ln PPV |  | 2.30 (1.82-2.91) | Odds Ratio ^a^ | <0.0001 |
|  | Surgery Type | Both / CABG | 2.35 (1.20-4.62) |  | 0.032 ^b^ |
|  |  | Valve / CABG | Not significant |  | 0.42 |
|  |  | Both / Valve | Not significant |  | NS |
| Ventilator Time > 11 hrs | Ln PPV | NA | 2.70 (2.13-3.44) | Odds Ratio ^a^ | <0.0001 |
|  | Psychotropics | Yes / No | 2.18 (1.39-3.43) |  | 0.0007 |
|  | Treatment | MM / Opioid | 1.80 (1.26-2.59) |  | 0.0014 |

^a^ Odds ratio for binary logistic regression, > 6 hours vs ≤ 6 hours ventilator time.

^b^ Note that while this is significant at α=0.05 threshold, it is not at a reduced threshold of 0.017.

Abbreviations: NA: Not applicable. GLM: General Linear Model. Ln: Natural log; PPV: Risk-adjusted Postoperative Prolonged Ventilation; CABG: Coronary Artery Bypass Graft Surgery; NS: Not significant; MM: Multimodal treatment group.
