# Supplementary figures and images for: The impact of a postoperative multimodal analgesia pathway on opioid use and outcomes after cardiothoracic surgery
Source: J Cardiothorac Surg. 2022 Dec 30;17:342. doi: 10.1186/s13019-022-02067-3 (PMC9801617; doi:10.1186/s13019-022-02067-3)

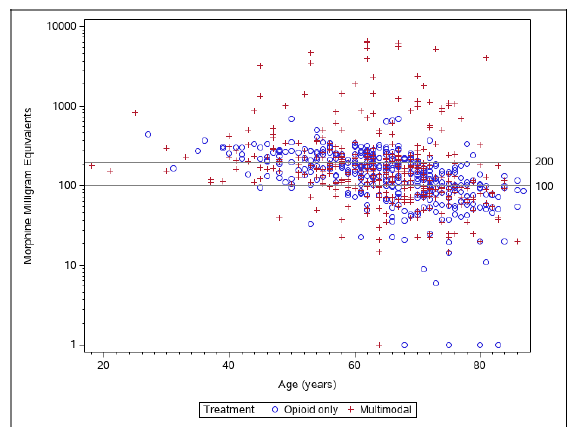

Supplement: Supplementary file 9 — Additional file 9. MME vs Age by Treatment Group. When MME is divided into 3 groups split at 100 and 200 mg, the treatment group is not significantly different. However, when MME is treated as continuous, it is significant because of outlier levels of MME. This is driven by differences in fentanyl use at the different institutions. All outliers > 700 mg are from fentanyl use. [file 13019_2022_2067_MOESM9_ESM.tif]

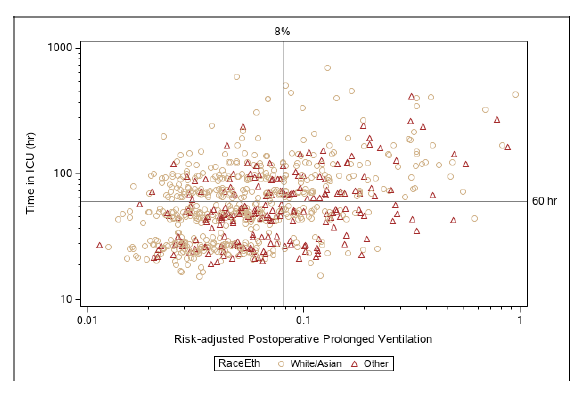

Supplement: Supplementary file 10 — Additional file 10. Time in ICU versus Postoperative Prolonged Ventilation by Race. White/Asian vs. African Amer. and Other. Results show that with a PPV less than 8%, African Americans and other races were significantly more likely to have an ICU time below 60-h, 75% vs 62%. [file 13019_2022_2067_MOESM10_ESM.tif]

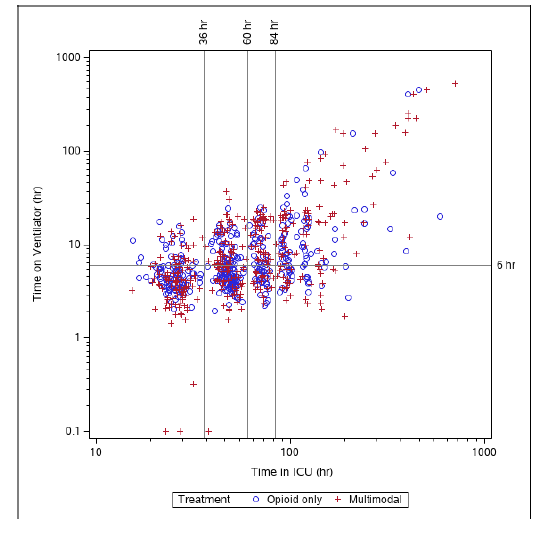

Supplement: Supplementary file 11 — Additional file 11. Scatterplot of Time on Ventilator vs Time in ICU by Treatment Group. Time on Ventilators was significantly correlated with Time in the ICU (p < 0.0001, r = 0.42, Spearman Rank Correlation). Because of this high correlation, only Time in ICU was used as an outcome. Breaks in ICU time at 36-h, 60-h, and 84-h (i.e., 1.5, 2.5, and 3.5 days) reflect lower discharge rates during the nighttime. Time in the ICU was dichotomized at 60-h for purposes of the regression analysis. Patients in the multimodal group had significantly longer times on the ventilator (p = 0.0061, Mann-Whitney U Test) and borderline longer time in the ICU (p = 0.058, Mann-Whitney U Test); however, these differences disappear from both regression models after adjusting for PPV and psychotropic use. For purposes of graphing on a log scale, 3 patients with no time on the ventilator were marked at 0.1-h. [file 13019_2022_2067_MOESM11_ESM.tif]

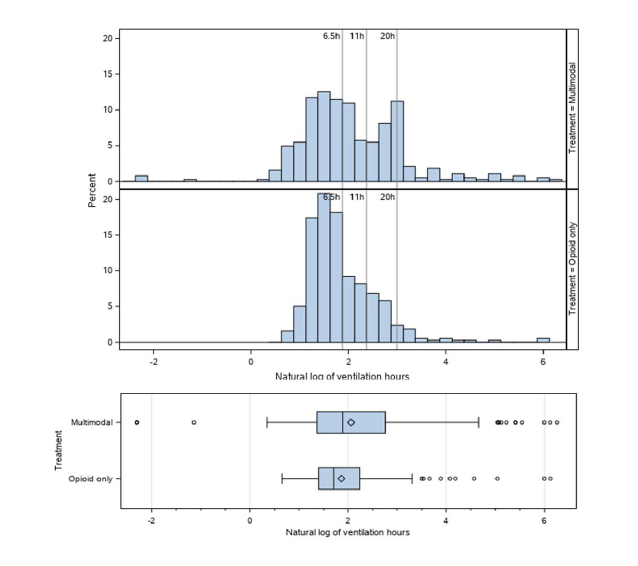

Supplement: Supplementary file 12 — Additional file 12. Histogram of Ventilation Hours by Treatment. Time on the ventilator is significantly higher in the multimodal analgesia group than the opioid only group (p = 0.0061, Mann-Whitney U Test). The histogram of the natural log of ventilator hours shows a positive skew in the opioid group. However, it is bimodal in the multimodal group, with a dip at 2.375, corresponding to 11-h, and a peak at 3.0, corresponding to 20-h. These unexpected results cannot be explained alone by the greater proportion of patients who had both CABG and valve surgeries (11% vs 6.6%). Note that for purposes of taking a natural log transformation, 3 patients without any time on the ventilator were marked as having spent 0.1-h, which translates to -2.3 on a log scale. [file 13019_2022_2067_MOESM12_ESM.tif]

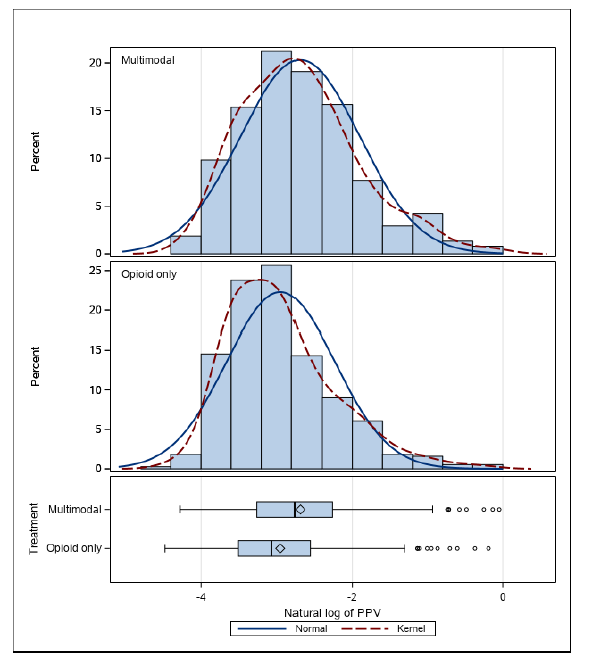

Supplement: Supplementary file 13 — Additional file 13. Histogram of Ln PPV by Treatment Group. The multimodal analgesia group was significantly more likely to have a higher risk-adjusted postoperative prolonged ventilation (PPV, p < 0.0001, Mann-Whitney U Test). Even after log transformation, the data are slightly positively skewed. However, the data were treated as if they were normally distributed for regression analysis purposes. [file 13019_2022_2067_MOESM13_ESM.tif]

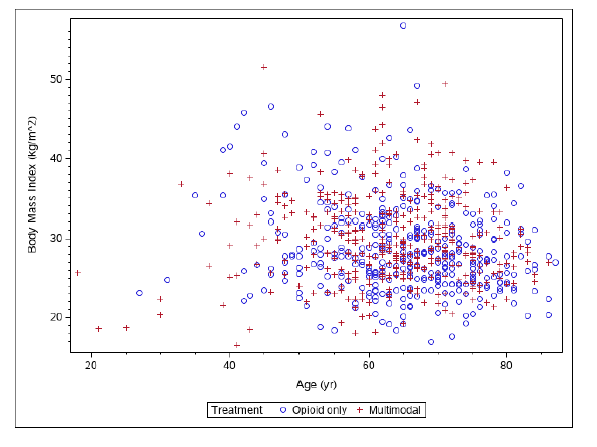

Supplement: Supplementary file 14 — Additional file 14. Scatterplot of Body Mass Index (BMI) vs Age by Treatment Group. Body mass index is significantly negatively correlated with age (p = 0.003, r = −0.13, Spearman Rank Correlation). Linear regression trend lines were not drawn because of outlier patients especially in the multimodal group with many younger, normal weight patients. This would have violated assumptions of the test. [file 13019_2022_2067_MOESM14_ESM.tif]

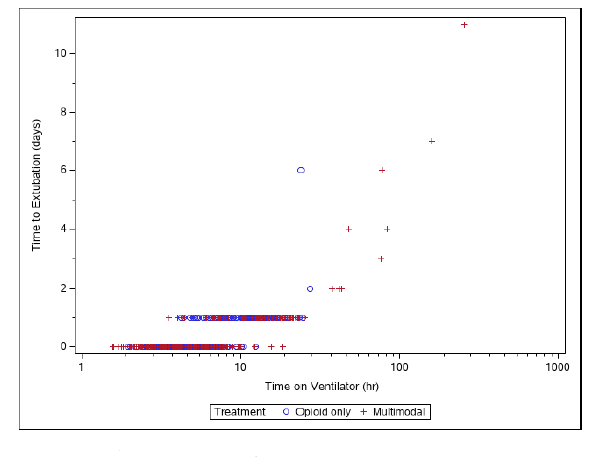

Supplement: Supplementary file 15 — Additional file 15. Scatterplot Time to Extubation vs Time on Ventilator by Treatment Group. Time to Extubation was highly correlated with Time on Ventilator (p < 0.0001, r = 0.73, Spearman Rank Correlation). Because of the high correlation, because Time to Extubation was only available for Coronary Artery Bypass Graft surgery patients, and because it was measured in days instead of hours, this variable was dropped as an outcome. Three patients with no time on the ventilator were omitted for purposes of graphing on a log scale. [file 13019_2022_2067_MOESM15_ESM.tif]

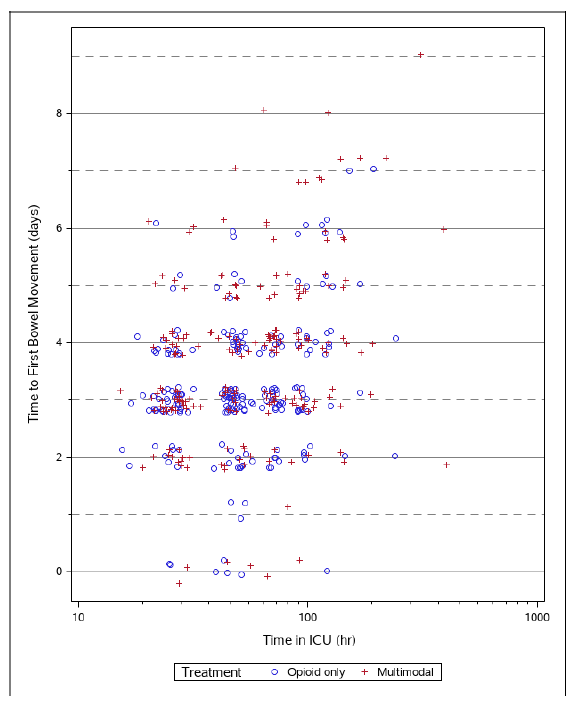

Supplement: Supplementary file 16 — Additional file 16. Scatterplot Time to First Bowel Movement (BM) vs Time in ICU. Time to first bowel movement (BM) was significantly correlated with time in ICU (p < 0.0001, r = 0.24, Spearman Rank Correlation). Data have been jittered about the BM axis to separate data points. [file 13019_2022_2067_MOESM16_ESM.tif]

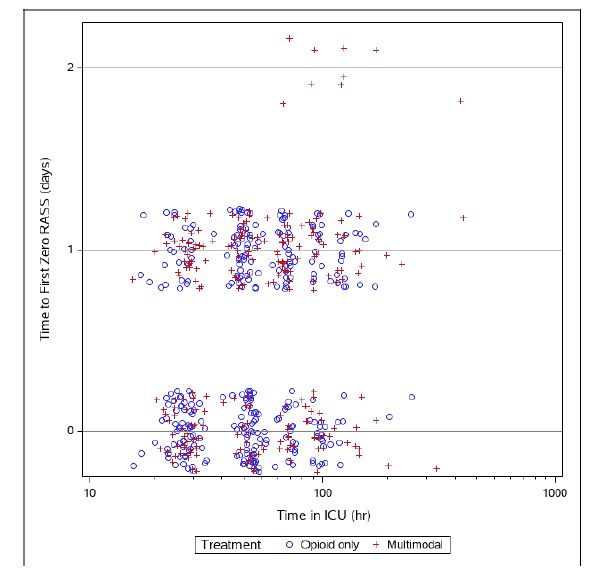

Supplement: Supplementary file 17 — Additional file 17. Scatterplot Time to First Zero RASS vs Time in ICU by Treatment. Results show a significant association of time to first zero score on the Richmond-Agitation-Sedation Scale (RASS) and Time in ICU (p = 0.0003, r = 0.15, Spearman Rank Correlation). Data have been jittered about the RASS axis to facilitate ease of viewing individual data points. [file 13019_2022_2067_MOESM17_ESM.tif]

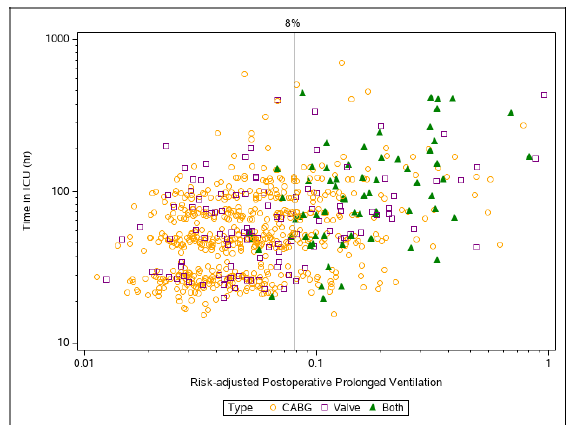

Supplement: Supplementary file 18 — Additional file 18. Scatterplot Time in ICU vs PPV by Surgery Type. Time in ICU was significantly correlated with Prolonged Postoperative Ventilation (PPV, p < 0.0001, r = 0.37, Spearman Rank Correlation). Both Time in ICU and PPV were significantly associated with surgery type (p < 0.0001, Kruskal Wallis Test). Ninety percent of patients who had "both" Coronary Artery Bypass Graft (CABG) and heart valve surgeries (green) had a PPV over 8% (grey reference line). [file 13019_2022_2067_MOESM18_ESM.tif]

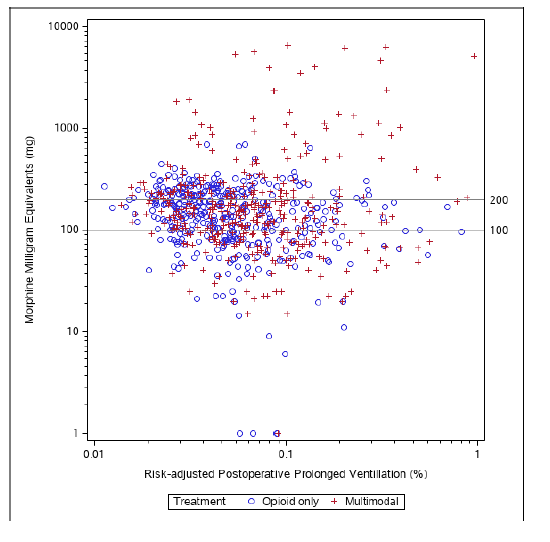

Supplement: Supplementary file 19 — Additional file 19. MME vs PPV by Treatment Group. Morphine Milligram Equivalents (MME) was significantly negatively associated with Prolonged Postoperative Ventilation (p < 0.0001, r = −0.16, Spearman Rank Correlation). Patients with MME > 700 mg in the MMA group were all given fentanyl. For purposes of graphing on a log scale, patients with no MME were marked at 1 mg. [file 13019_2022_2067_MOESM19_ESM.tif]

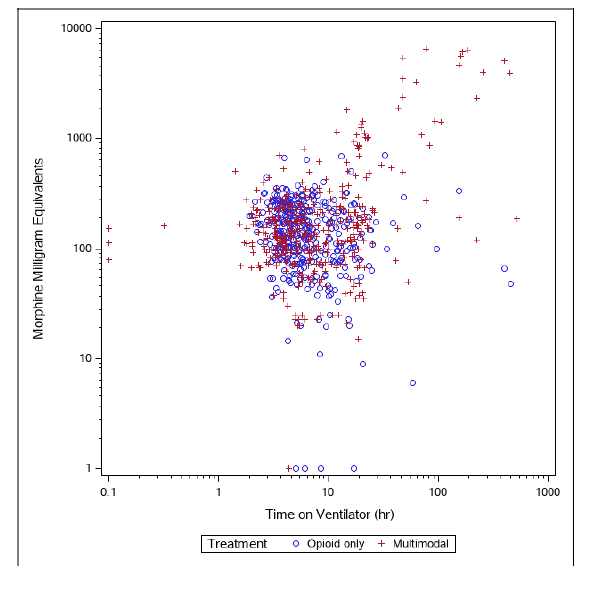

Supplement: Supplementary file 20 — Additional file 20. MME vs Time on Ventilator by Treatment Group. Morphine Milligram Equivalents (MME) was not significantly associated with Time on Ventilator (p = 0.45), Spearman Rank Correlation) nor with Time in ICU (data not show, p = 0.17). However, patients with MME > 700 mg in the MMA group, who were all given fentanyl, all had longer ventilator times of > 10-h. But patients with long time on the ventilator did not all have outlier levels of MME. For purposes of graphing on a log scale, patients with no MME were marked 1 mg and patients with no ventilator time were marked at 0.1-h. [file 13019_2022_2067_MOESM20_ESM.tif]
