# Supplementary material for: The impact of a postoperative multimodal analgesia pathway on opioid use and outcomes after cardiothoracic surgery
Source: J Cardiothorac Surg. 2022 Dec 30;17:342. doi: 10.1186/s13019-022-02067-3 (PMC9801617; doi:10.1186/s13019-022-02067-3)
Supplement: Supplementary file 21 — Additional file 21. Summary of Supplemental Data. [file 13019_2022_2067_MOESM21_ESM.docx]

**Supplemental Material**

The Impact of a Standardized Multimodal Pain Management Program

On Opioid Use and Outcomes after Cardiac Surgery

Ceressa T. Ward et al, 2022

# **Association of Confounders**

See Table S1.

Because of the high correlation between PPV, OMM and OM (r = 0.88-0.97), only PPV was used in models. PPV was correlated with both Age and BMI. Age was negatively correlated with BMI.

See Table S2.

Nonwhites had higher PPV, lower age, lower BMI, more females and fewer patients on psychotropics. Females had higher PPV, were more likely to have valve surgery, and more likely to be on 2-4 adjuncts. Patients having value surgery were younger, more likely to be on 2-4 adjuncts, and more likely to be on psychotropics. Patients having both CABG and Valve surgeries were older and had higher PPV. Patients on 2-4 adjuncts were heavier and had higher PPV. Patients on psychotropics did not have statistically significant differences in PPV, age or BMI.

# **Details of Outcome Selection and Grouping**

Initially there were eight outcomes of interest: MME, Ambulation, Confusion Assessment Method (CAM-ICU for delirium), Time to Extubation, Time on Ventilation, Time in the Intensive Care Unit (ICU), Time to First Bowel Movement, and Time to zero score on Richmond Agitation Sedation Scale (RASS). Data for Ambulation, CAM-ICU Delirium, Time to Extubation and Time to first 0 RASS score were only available for patients who had had only Coronary Artery Bypass Graft (CABG) surgery, while data for Time to First Bowel Movement was only available for a portion of CABG surgeries.

CAM-ICU had to be dropped because only ten of the 552 patients had delirium, which is too few to establish differences. The remaining seven outcomes were examined if any were correlated, to see if they could be examined collectively first, to reduce the greater risk of false positives caused by multiple testing. Table S3 shows the Spearman Rank Correlation of the six continuous outcomes, along with how they were eventually grouped or transformed for analysis. Extubation and Ventilation Time were closely correlated (r=0.73). Data were missing for 212 patients for Extubation, and the measure for Extubation was less refined (days versus hours), so Extubation was dropped. Time in the ICU and Time on Ventilator were also correlated (r=0.42). Time in the ICU dichotomized naturally at 60 hours, so Time on Ventilator was also dropped. While Time to first 0 RASS score and Time to First Bowel Movement post-surgery were both correlated with Time in ICU, they were not significantly correlated with each other. Ambulatory status was not significantly associated with any other outcome (Mann-Whitney U Test).

MME, Time in ICU and risk-adjusted Postoperative Prolonged Ventilation (PPV) were strongly positively skewed. The natural log transformation of MME was slightly leptokurtic (too narrow), but adequate to assume normal distribution. The log transformation of Time in ICU and PPV were somewhat positively skewed, but also adequate to assume normal distribution. Formal tests of normality, such as the Shapiro-Wilks Test, are generally considered too conservative, rejecting the null hypothesis too frequently for larger sample sizes, so they were not conducted. Comparison of multiple regression models with the ICU either log transformed or grouped gave similar results.

Table S4 shows preliminary analysis of these outcomes by treatment group, unadjusted for other confounders, along with how they were grouped or removed for final analysis. Statistical significance or absence cannot be meaningfully accepted from this table, given the large differences in the two groups seen in important confounders, including PPV. MME and Ambulatory Status were analyzed alone, since they were not significantly correlated with other outcomes. Time in ICU, Time to First Bowel Movement and Time to First Zero RASS Score post-surgery were grouped and analyzed together first as a group. If this were shown to be statistically significant, then they would be analyzed separately, similar to how if ANOVA is significant, then *post hoc* analysis of which groups are different is permissible.

In assessing how to analyze the 3 time variables together, we had to consider the type of variable. While Time to First Zero RASS score is technically continuous, it is effectively binary with values of 0 or 1 day, with only 9 patients (1.6%) at 2 days. Therefore, this variable was grouped as 0 days vs. 1-2 days for all analyses. Similarly, Time to First Bowel Movement is more ordinal than continuous, with a median 3 days, and an IQR of 3-4 days. Therefore, this variable was dichotomized at 0-3 days vs. 4-9 days, with the latter representing constipation. Time in ICU is continuous, but positively skewed and multimodal with natural dips occurring at about 36, 60 and 84 hours, corresponding to lower discharge rates late at night (i.e. 1.5 days, 2.5 days, and 3.5 days). Dichotomizing the ICU data at 60 hours was chosen for the joint time analysis, to give roughly identical groups (58% vs. 42%), so all three outcomes would be binary. In the joint analysis with grouped variables, only patients with data for all three variables could be included, so comparisons of the full set versus the partial set with BM data only (N=428) are presented in Table S4. The proportions in the full data set and in the partial set of patients are almost identical for both outcomes, indicating low potential for selection bias within the outcomes.

# **MME Grouped**

When MME is log transformed and treated as a continuous variable, the Multimodal group is a significant predictor of MME, which is opposite of our hypothesis. See Table 3 in the main text. However, as seen in Table S5 below, if MME is grouped, the Treatment group is not significant. This is because outliers in the Multimodal group, caused by the greater use of fentanyl, are driving results. See also Figure S1.

# **ICU Time and Race**

Patients who were not white or Asian (i.e. African Americans and others) were twice as likely to have ICU times less than 60 hours. For further analysis, PPV was dichotomized at 8%, which was selected because 90% of patients with both CABG and valve surgeries were above 8%. Results in Table S6 and Figure S2 show that African Americans and Others were significantly more likely than Whites or Asians to have shorter ICU time if their disease severity was low, below 8%. This was not statistically significant when their PPV was above 8%, using the adjusted alpha of 0.017. Race/Ethnicity was not a significant predictor of time on a ventilator. Almost all patients in this sample who were not white or Asian American were African American (89%).

# **Time on Ventilator**

Time on the ventilator was significantly correlated with time in the Intensive Care Unit (ICU) (p<0.0001, r=0.42, Spearman Rank Correlation). Time in the ICU was chosen over Time on the Ventilator because it naturally dichotomized at 60 hours (Figure S3).

There were some interesting trends in the distribution of the natural log of time on the ventilator. While the distribution was slightly positively skewed for the opioid-only group, it was bimodal for the multimodal analgesia group. This occurs with a dip at the left edge at 2.375 and peaks at 3.0. This corresponds to 11 hours and 20 hours, respectively. See Figure S4. Recall that treatment groups are at different institutions.

Patients who had both CABG and valve surgery were more likely to be in the multimodal analgesia group (11% vs 6.6%). However, as seen in Table S7, patients in the MA group who only had CABG surgery were also significantly more likely to spend more than 11 hours on the ventilator (30% vs 15%). In contrast, there were no significant differences in time on the ventilator by treatment group for patients who had had valve or both valve and CABG surgeries.

A regression of time on the ventilator was conducted, to confirm that results were parallel to time in the ICU. As seen in Table S8 below, predictors were similar when time on the ventilator was treated as a continuous, log-transformed variable, with PPV and psychotropics significant predictors. When time on the ventilator was dichotomized at 6 hours, to give roughly equal groups, psychotropics was replaced by surgery type. Only the comparison of Both to CABG only surgeries was statistically significant. Removing surgery type from the dichotomized model raises the odds ratio of Ln PPV to 2.58 (95% CI 2.06-3.22). When ventilator time was split at 11 hours, the treatment group became statistically significant, with those in the multimodal group having a significantly greater chance of having a ventilator time greater than 11 hours. Global p-values for all models were p<0.0001.

Because Time on the Ventilator was omitted as an outcome in the initial steps, results present here cannot be considered statistically “official.” They are presented primarily to show similarities and differences to time in the ICU.

# 
